# Identifying progressive CKD from healthy population using Bayesian network and artificial intelligence: A worksite-based cohort study

**DOI:** 10.1038/s41598-019-41663-7

**Published:** 2019-03-25

**Authors:** Eiichiro Kanda, Yoshihiko Kanno, Fuminori Katsukawa

**Affiliations:** 1 Medical Science, Kawasaki Medical School, Okayama, Japan; 2 Department of Nephrology, Tokyo Medical University, Tokyo, Japan; 3 Sports Medical Research Center, Keio University, Kanagawa, Japan

## Abstract

Identifying progressive early chronic kidney disease (CKD) patients at a health checkup is a good opportunity to improve their prognosis. However, it is difficult to identify them using common health tests. This worksite-based cohort study for 7 years in Japan (n = 7465) was conducted to evaluate the progression of CKD. The outcome was aggravation of the KDIGO prognostic category of CKD 7 years later. The subjects were male, 59.1%; age, 50.1 ± 6.3 years; and eGFR, 79 ± 14.4 mL/min/1.73 m^2^. The number of subjects showing CKD progression started to increase from 3 years later. Vector analysis showed that CKD stage G1 A1 was more progressive than CKD stage G2 A1. Bayesian networks showed that the time-series changes in the prognostic category of CKD were related to the outcome. Support vector machines including time-series data of the prognostic category of CKD from 3 years later detected the high possibility of the outcome not only in subjects at very high risks but also in those at low risks at baseline. In conclusion, after the evaluation of kidney function at a health checkup, it is necessary to follow up not only patients at high risks but also patients at low risks at baseline for 3 years and longer.

## Introduction

In Japan, the number of chronic kidney disease (CKD) patients was estimated to be 13.3 million in 2005^1^. And the number of end-stage renal disease (ESRD) patients was 324986 in 2015^2^. With the aging of the Japanese population, the number of CKD patients is estimated to continue to increase.

CKD has been reported to be a risk factor for death, ESRD, and cardiovascular disease (CVD) in Japan^3,4^. The number of patients with ESRD due to diabetic kidney disease and nephrosclerosis, which are associated with aging, has been increasing^2^. The prognosis of CKD patients can be improved by identifying such patients at CKD stages G1 and G2 and implementing therapeutic strategies to reduce the incidence of CVD events and ESRD. Clinical practice guidelines established by the Japanese Society of Nephrology (JSN) and American College of Physicians (ACP) recommend screening for CKD^1,5^.

CKD stages are determined on the basis of the estimated glomerular filtration rate (eGFR) and proteinuria grade^1,6^. Considering the relationship between CKD stages and patients’ CKD prognosis, the prognosis is classified into four categories according to risk from low (green) to very high (red)^1,6^. These prognostic categories of CKD are guides for CKD patients to be treated and referred to nephrologists^1,6^. However, the rate of referral to nephrologists on the basis of the prognostic categories of CKD was low^7^.

One of the reasons for the difficulty in treating CKD is that the decline in eGFR is slower in early CKD stages than in late CKD stages, and a long follow-up period is required^8^. Moreover, the association among many causes of CKD progression such as hypertension, diabetes mellitus (DM), and dyslipidemia is complex^1,6,9,10^. The treatment strategy for early CKD has not been fully established yet.

If CKD patients at high risks of CKD progression are identified at CKD stages G1 and G2, who are usually diagnosed as being at low risks, and their lifestyles are improved, the progression of their CKD will be prevented. A health checkup is a good opportunity to identify such patients from a healthy population. Therefore, to identify CKD patients at high risks of CKD progression, and to utilize the results at health checkups, the aims of this study were to (1) evaluate time-series changes in CKD stage, (2) determine the risk factors for CKD progression using Bayesian networks, and (3) identify CKD patients at high risks of CKD progression using support vector machine (SVM) models and data of common tests from a longitudinal worksite-based study of health checkup in Japan.

## Results

### Baseline characteristics

The baseline characteristics including biochemical data in 2009 are shown in Table 1. Regarding CKD stages, G2 Proteinuria grade (P) (−) and G1 P(−) were mostly observed (Table 2). The CKD stages from 2009 to 2016 showed similar distributions (data not shown). On the basis of the prognostic categories of CKD, 6436 (86.2%) subjects were at low risk; 730 (9.8%), moderately increased risk; 251 (3.4%) high risk; and 48 (0.6%), very high risk. Among the subjects with data of their CKD stages in 2009 and 2016 (n = 3927), 3327 (84.7%) were at low risk; 509 (13.0%), moderately increased risk; 68 (1.7%), high risk; and 23 (0.6%), very high risk. The outcome was observed in 441 (11.2%).

**Table 1:** Baseline characteristics of subjects with data.

Variables	Values
Age	50.1 ± 6.3
Male (%)	4793 (64.2)
Hypertension (%)	2235 (29.9)
DM (%)	421 (5.6)
Dyslipidemia (%)	2661 (35.7)
BMI (kg/m^2^)	23.3 ± 3.3
Waist circumference (cm)	82.7 ± 8.9
Systolic blood pressure (mmHg)	123.6 ± 16.9
Diastolic blood pressure (mmHg)	78.8 ± 11.8
Casual blood glucose (mg/dL)	94.6 ± 17.8
HbA1c (NGSP) (%)	5.6 ± 0.6
Serum LDL cholesterol level (mg/dL)	124.1 ± 31.4
eGFR (mL/min/1.73 m^2^)	79 ± 14.4

Variables are expressed as number, or mean ± standard deviation.

Abbreviations: DM, diabetes mellitus; BMI, body mass index; LDL, low-density lipoprotein; eGFR, estimate glomerular filtration rate.

**Table 2:** Distribution of CKD stages.

	A1	A2	A3	A3
	(−)	(±)	(+)	(2+)
G1	1375 (18.4)	53 (0.7)	25 (0.3)	10 (0.1)
G2	5061 (67.8)	355 (4.8)	129 (1.7)	36 (0.5)
G3a	322 (4.3)	34 (0.5)	18 (0.2)	16 (0.2)
G3b	17 (0.2)	3 (0.0)	2 (0.0)	9 (0.1)

Values are numbers of subjects (%). (−), (±), (+), and (2+) show proteinuria grades.

### Time-series changes in CKD stage

The comparison of CKD stages between 2009 and any of the following years was examined. Most of the subjects showed a stable CKD stage, and some of them showed that their GFR increased or decreased. From 2009 to 2010, most of the subjects in G1 P(−) (70.8%) and G2 P(−) (81.4%) showed a stable CKD (Supplementary Fig. S1), whereas 22.8% of the subjects in G1 P(−) in 2009 were in G2 P(−) in 2010. On the other hand, 10.6% of the subjects in G2 P(−) in 2009 were in 2010 G1 P(−), and 2.3% were in G3 P(−) in 2010.

**Figure 1:**
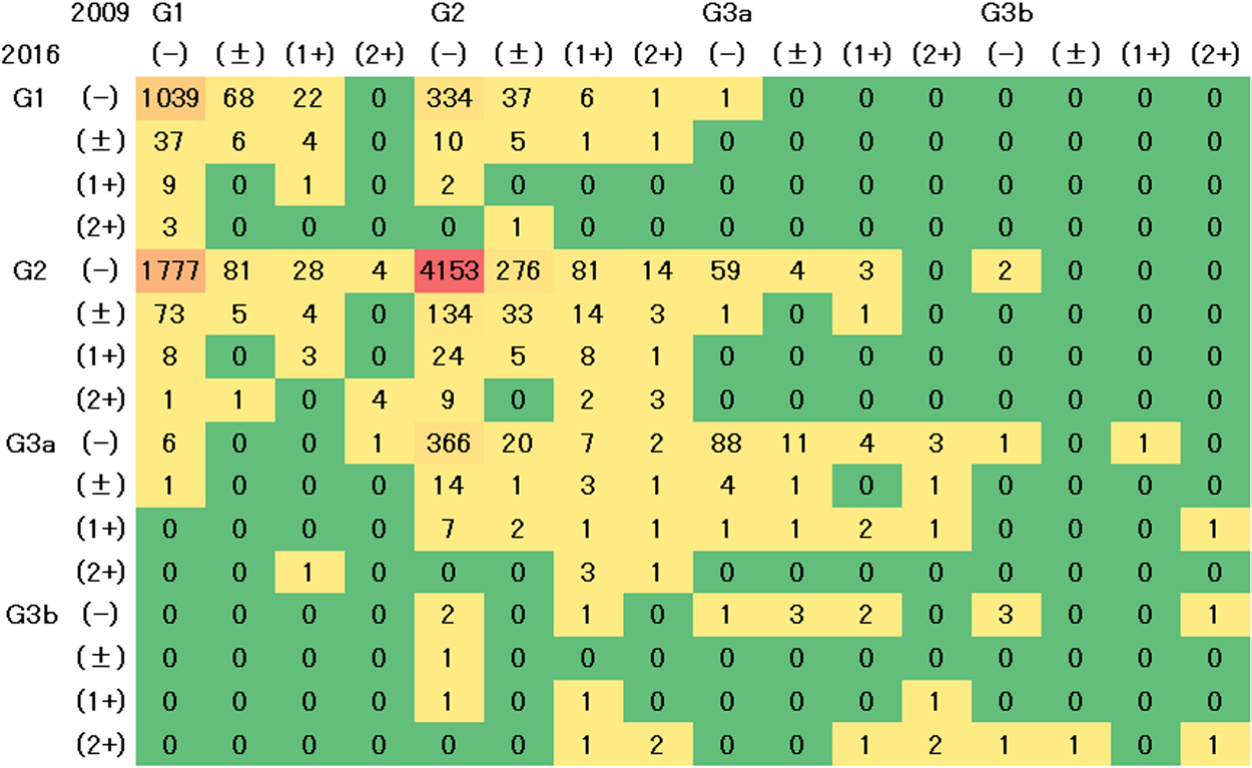
Change in distribution of CKD stages from 2009 to 2016. The distribution was analyzed using data of subjects with CKD stages in 2009 and 2016 (n = 8991). Values show the number of subjects by CKD stage. G1 to G3b and (−) to (2+) are GFR categories of CKD stages, and proteinuria grades, respectively. Abbreviations: CKD, chronic kidney disease; GFR, glomerular filtration rate.

The changes in the distribution of CKD stage from 2009 to any of the following years showed similar tendencies (Supplementary Fig. S2 and Fig. 1). The number of subjects whose CKD stage changed from G2 P(−) to G3a P(−) tended to increase from 2012 (Supplementary Fig. S2). From 2009 to 2016, most of the subjects in G1 P(−) (35.2%) and G2 P(−) (82.1%) showed stable CKD (Fig. 1), whereas 60.2% of the subjects in G1 P(−) in 2009 were in G2 P(−) in 2016. On the other hand, 6.6% of the subjects in G2 P(−) in 2009 were in G1 P(−), and 7.2% were in G3a P(−) in 2016.

**Figure 2:**
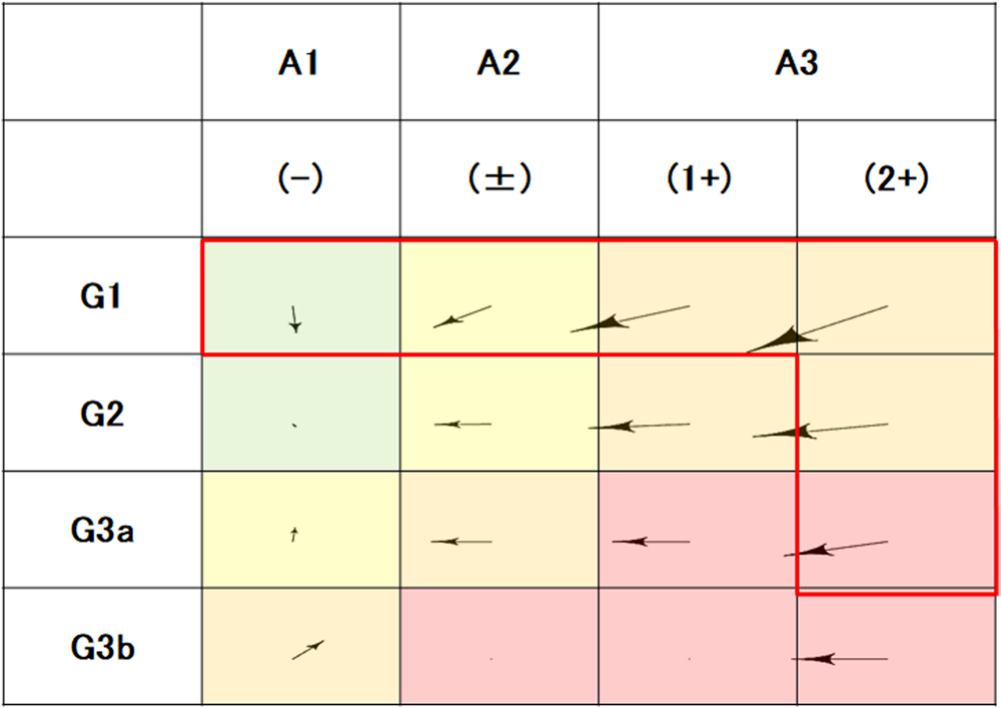
Mean changes in CKD stages of subjects from 2009 to 2016. G1 to G3b and (−) to (2+) are GFR categories of CKD stages and proteinuria grades, respectively. Colors of cells mean low (green), moderately increased (yellow), high (orange), and very high risks (red) as the KDIGO prognostic categories of CKD. Arrows show the mean direction of changes in CKD stages of participants from 2009 to 2016. A red line surrounds CKD stages with high risks. Abbreviations: CKD, chronic kidney disease; GFR, glomerular filtration rate.

Vector analysis showed that any G1 stages and from G2 to G3a with P(2+) tended to show the progress of GFR categories (Fig. 2). In most of the CKD stages except G3b P(−), the proteinuria grade decreased. And G1 P(−) tended to be more progressive than G2 P(−).

### Increase in severity of CKD and related factors

**Figure 3:**
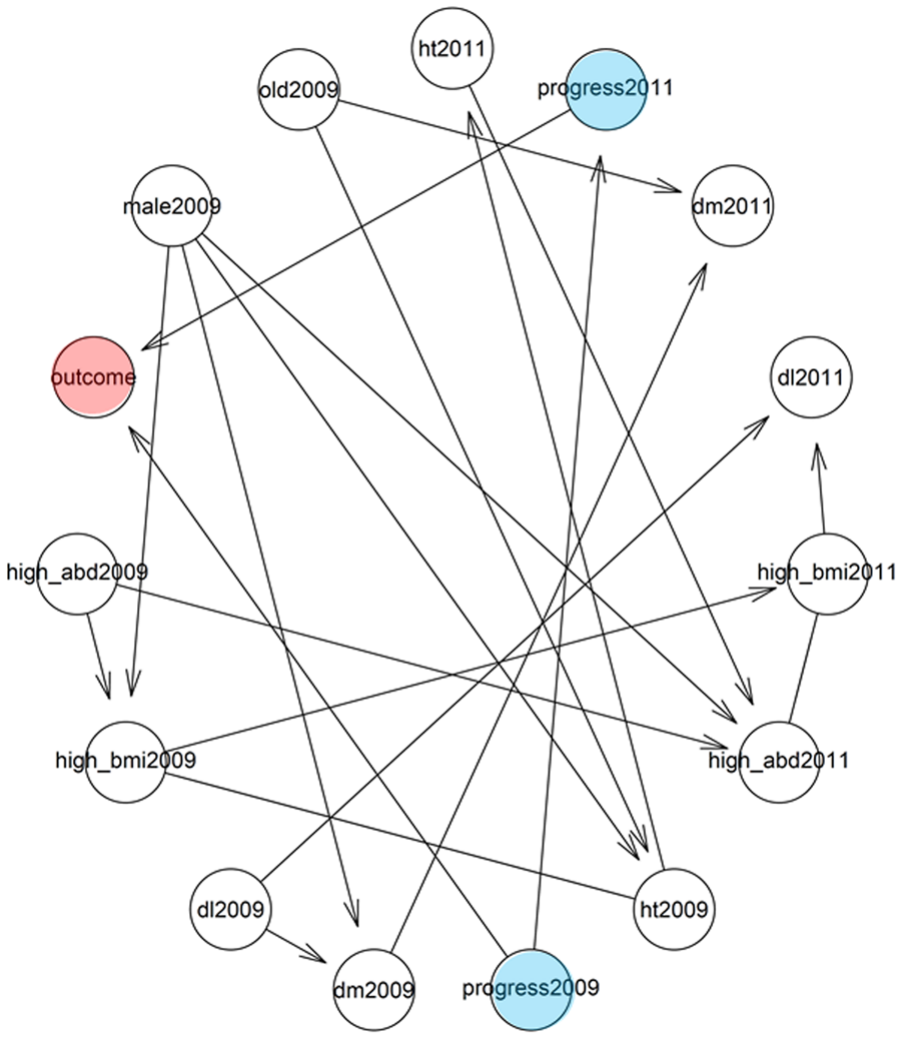
Bayesian network constructed using the data in 2011. Arrows show the causal relationships between variables. Abbreviations: Outcome, the progression of the prognostic category of CKD or very high risk in 2016; progress2009, the prognostic category of CKD in 2009; ht2009, hypertension in 2009; dm2009, diabetes mellitus in 2009; dl2009, dyslipidemia in 2009; old2009, age of 46 years or more in 2009; high_BMI, body mass index of 22.8 kg/m^2^ or more in 2009; high_abd, waist circumference of 81.4 cm or more in 2009.

Using the time-series data of two points (2009 and any of the following years), the Bayesian network showed causal relationships between variables. It showed that the outcome was affected by the prognostic categories of CKD in 2009 and 2010, and the presence of hypertension in 2009 (Supplementary Fig. S3). In each of the Bayesian networks in 2009 and 2011 to 2015, the outcome was affected by the prognostic category of CKD in 2009, and that in each year from 2011 to 2015, but not by other variables (Fig. 3). It was suggested that the time-series data of the prognostic category of CKD were useful variables for the prediction of the outcome.

### Prediction of increase in severity of CKD

The SVM models predicted the progression of the prognostic category of CKD (Table 3). The test errors of Models 2009 + 2012 to 2009 + 2016 were smaller than that of Model 2009 + 2011.

**Table 3:** Accuracy of prediction of progression of prognostic categories of CKD from 2009 to 2016 using SVM.

SVM models	Training error	Test error
Model 2009	0.124007	0.1205273
Model 2009 + 2010	0.123015	0.1211551
Model 2009 + 2011	0.124007	0.1205273
Model 2009 + 2012	0.123006	0.1186441
Model 2009 + 2013	0.122008	0.1186441
Model 2009 + 2014	0.125008	0.1192718
Model 2009 + 2015	0.123012	0.1186441

SVM models include the prognostic categories of CKD in 2009 and each following year.

Abbreviations: SVM, support vector machine; training error, cross validation error of accuracy to predict the outcome using the training dataset; test error, error of accuracy to predict the outcome using the test dataset.

**Figure 4:**
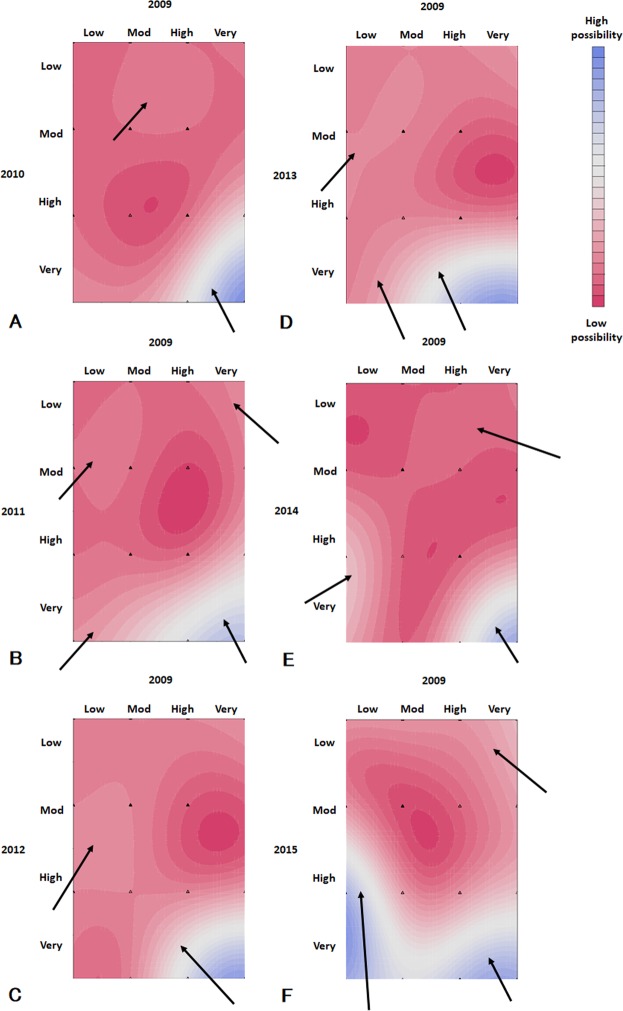
Heat map for predicting the outcome from 2009 to 2016. A heat map shows the possibility of the outcome estimated using SVM models on the basis of data at two points in 2009 and any of the following year. Blue and red areas indicate high and low risks, respectively. Arrows show the high-possibility area of the outcome. (**A**) Data 2009 and 2010. (**B**) Data 2009 and 2011. (**C**) Data 2009 and 2012. (**D**) Data 2009 and 2013. (**E**) Data 2009 and 2014. (**F**) Data 2009 and 2015. Abbreviations: Low, low risk of the prognostic categories of CKD; Mod, moderately increased risk; High, high risk; Very, very high risk.

The heat maps showed the possibility of the outcome as determined using the SVM models (Fig. 4, Supplementary Fig. S4). In the SVM Model 2009 + 2010, the area for subjects at very high risks in 2009 and 2010 indicated a high possibility of the outcome (Fig. 4A). SVM models showed the different distributions of the probabilities of the outcome from the expected ideal probabilities (Supplementary Fig. S4G). From Model 2009 + 2011, the area for the subjects with a high possibility of the outcome was observed in the subjects at low risks in 2009 (Fig. 4B, Supplementary Fig. S4B). Model 2009 + 2012 showed that the subjects at moderately or high risks in 2009 showed a high possibility of the outcome (Fig. 4C). From Model 2009 + 2013, the area for the subjects with a high possibility of the outcome expanded to the area for the subjects at low risks in 2009 (Fig. 4D–F, Supplementary Fig. S4D–F). The subjects at low risks in 2009 and high or very high risk in 2014 showed a high possibility of the outcome (Fig. 4E, Supplementary Fig. S4E). This trend was enhanced in the Model 2009 + 2015 (Fig. 4F, Supplementary Fig. S4F).

## Discussion

In this study, we investigated the time-series changes in the distribution of CKD stages, and it showed that the number of subjects showing CKD progression started to increase from 3 years later. The vector analysis showed the trends of CKD progression in each CKD stage; CKD stage G1 P(−) was more progressive than CKD stage G2 P(−). The Bayesian networks showed that the time-series changes in the prognostic category of CKD were related to the outcome. Support vector machines including time-series data of the prognostic category of CKD from 3 years later detected the high possibility of the outcome not only in subjects showing very high risks but also in those showing low risks at baseline. These results using our methods have never been reported as far as we searched the literature.

In this study, we evaluated a healthy population and the time-series changes in their CKD stage. The majority of the subjects were in G2 P(−) and G1 P(−) in each year, which was in accordance with previous studies^11,12^. Among the subjects in G2 P(−), their improvement to G1 P(−) was more commonly observed than their progression to G3 P(−) over 2 years. Then, the number of subjects showing the CKD progression gradually increased from 3 years later. The poor reproducibility of proteinuria and eGFR is often observed^5^. This phenomena of exacerbation and improvement of CKD stage might make it difficult to diagnose CKD at an early stage, and to identify CKD patients at high risks of CKD progression. There has been no trajectory study of changes in early CKD stages based on the prognostic categories of CKD as in this study, to the best of our knowledge^5^.

Proteinuria and eGFR have been used as markers for monitoring the clinical course of CKD^1^. Proteinuria is an appropriate marker for detecting kidney diseases such as glomerular nephritis, and diabetic nephropathy. However, as in this study, most of the subjects from a healthy population do not have proteinuria; thus, the use of proteinuria as a marker is limited. On the other hand, an eGFR change of more than 30% has been proposed as a surrogate endpoint of ESRD^13,14^. The relationship between eGFR change and the risk of ESRD was validated in subjects with eGFRs of more than 60 mL/min/1.73 m^2^ using health checkup data in Okinawa, Japan^15^. In the Okinawa study, the risk of ESRD is associated with not only a decrease in eGFR but also an increase in the extent of eGFR change^15^. Because the increase in eGFR does not always indicate the improvement of kidney function, care is necessary in the use of eGFR change as a surrogate endpoint of ESRD. Thus, in CKD stages G1 to G3, either proteinuria or eGFR is not sufficient for evaluating kidney function; both of them are required. The prognostic category of CKD, which includes both proteinuria and eGFR, is a candidate index for evaluating CKD progression^1,6^. In this study, the vector analysis showed the trends of CKD progression in each CKD stage, and we found that CKD stage G1 P(−) was more progressive than CKD stage G2 P(−). These results suggest a possibility that CKD progression is a function of eGFR and proteinuria, that eGFR and proteinuria are associated with each other, and that there is a limitation in treating eGFR and proteinuria independently. Thus, there is a need to consider the complex relationships between factors related to CKD progression when establishing models to estimate the possibilities of the outcome. From these observations, Bayesian networks, which can treat the relationships between the factors, were used in this study.

Here, the analyses using Bayesian networks showed that the prognostic categories of CKD at the start and the following years were associated with the aggravation of CKD. Moreover, the analysis using the SVM models including time-series data of the prognostic category of CKD from 3 years later could predict the high possibility of the outcome not only in subjects showing very high risks but also in those showing low risks at baseline. Even if a subject was at a low risk at baseline, this low risk was not guaranteed over a long period. These results suggest that it is necessary to follow up not only patients showing high risks but also those showing low risks at baseline.

Yearly evaluation of the prognostic category of CKD by health checkup is recommended by the JSN CKD guideline^1^. Then, how long should the results of health checkup be followed up to identify CKD patients at high risks of the CKD progression? The Okinawa study showed that at least 3 years is required to observe the relationship between the eGFR change and the risk of ESRD^15^. In the present study, from 3 years later, the number of CKD patients gradually increased and the accuracy of SVM models increased. From 4 years later, the heat maps of SVM models indicated the subjects at low risks at baseline and high possibility of the outcome. These results suggest that depending on the characteristics of the study population, the observation period to accurately evaluate the CKD progression should be at least 3 years.

The analysis using the Bayesian network showed that the CKD progression was associated with the existence of hypertension at baseline. These results suggest that hypertension is a risk factor for the CKD progression. This is in accordance with previous studies^1,3,6,9^. These lines of evidence suggest that one of the causes of the CKD progression might be atherosclerosis, which leads to nephrosclerosis. A prospective cohort study showed that the risk factors for incident CKD are hypertension, aging, DM, and dyslipidemia, which are also associated with atherosclerosis^9^. In the present study, although DM and dyslipidemia were not associated with the outcome, when these comorbid conditions are observed, they should be treated appropriately.

The results of this study and the JSN and Kidney Disease, Improving Global Outcomes (KDIGO) guidelines suggest the usefulness of the prognostic categories of CKD for the screening for CKD patients showing high risks^1,6^. Considering these findings, after the evaluation of kidney function at a health checkup, it is necessary to follow up not only patients showing high risks but also those showing low risks at baseline for 3 years and longer. Moreover, (1) when a subject is diagnosed to be at high or very high risk, (2) when comorbid conditions, such as hypertension, DM, and dyslipidemia, are found, and (3) when the prognostic category of CKD progresses from low risk at baseline to very high risk three years after or later, it would be better to examine the causes of CKD, review current management, and consider referring a patient to a nephrologist^1,6^. These steps from health checkup to treatment make it possible to provide careful medication that meets CKD patients’ needs. The promotion of health checkup based on the prognostic category of CKD may be useful for establishing public-health policies to decrease the prevalence of CKD.

This study has several limitations. First, because of the observational nature of this study, the results may be biased by unmeasured confounders. Second, the population mainly consisted of healthy workers, and did not include elderly people and the subjects with missing data in this study. Moreover, this study was carried out in only one region in Japan. These might have caused selection bias. More subjects recruited from all over Japan would be better to prevent selection bias. Third, age is associated with eGFR and the progression of CKD. The age of the subjects might affect the results. Moreover, because not only age but also other characteristics might affect the results of this study, although many models (Bayesian networks and SVM models) were developed and integrated to infer universal results using many sampling datasets based on the boot strapping method, cohort studies of populations with characteristics different from those in this study such as age, gender, and location might be required to show the external validity. Fourth, the data were not sufficient for assessing true outcomes such as events of death, ESRD, and CVD, and various factors associated with CKD progression such as comorbid conditions, and medications. The effects of these factors on CKD will be evaluated in our future studies. Moreover, it has been reported that the risk of ESRD in a healthy population (eGFR more than 60 ml/min/1.73 m^2^) was only 186 (0.32%) in 58,292 persons over a 15 year period^14^. It is very difficult to use ESRD as the true end point in cohort studies of patients in CKD stages G1 to G3. Therefore, in this study, the outcome was defined as the progression of a prognostic category of CKD or the high risk at the end of this study. Fifth, in this study, the patients were followed up for 7 years, which may not be enough to evaluate a true endpoint such as ESRD. However, the “Guidelines for clinical evaluation of chronic kidney disease” indicate that 3 years of observation is appropriate for evaluating eGFR changes in a healthy population; therefore, 7 years might be enough to observe changes in kidney function at the individual level^14^. Sixth, accuracies of the prediction of the outcome of SVM models were not compared with those of other prediction models. SVM was selected not only for accurate prediction but also for the evaluation of the effects of variables on patients’ prognosis. Multivariate SVM models and deep learning models will be needed for more accurate predictions. To apply the machine learning models in clinical settings, social implementation such as software development may be useful.

In conclusions, this study showed that the progression of CKD in a healthy population is associated with the time-series changes in the prognostic category of CKD. After the evaluation of kidney function at a health checkup, it is necessary to follow up not only patients showing high risks but also those showing low risks at baseline for 3 years and longer.

## Methods

### Dataset

This study was an observational and worksite-based study conducted in Yamagata, in the northern part of Japan. This study was approved by the ethics committees of Sports Medical Research Center of Keio University, and was exempt from the need to obtain informed consent from participants (No. 2013-06). The study was performed in accordance with the relevant guidelines and the Declaration of Helsinki.

In this study, we analyzed data collected every year from the medical checkup records of asymptomatic people working at Yamagata Municipalities Mutual Aid Association from 2009 to 2016. The study population consisted of 16734 subjects. Subjects with data on serum creatinine level were included in this study (n = 13946) (Supplementary Fig. S5). Those with missing data on baseline characteristics were excluded from this study. Finally, 7465 subjects were included in the study.

The baseline patient data included age, gender, body mass index (BMI), waist circumference, systolic and diastolic blood pressures, casual blood glucose, hemoglobin A1c (HbA1c) (NGSP), serum low-density lipoprotein (LDL) cholesterol, and creatinine levels, and proteinuria grade (dip stick). Because subjects with much proteinuria more than (2+) were very rare, they were assigned a grade of (2+). eGFR was calculated using the following equation for the Japanese population^16^:

$${\rm{eGFR}}\,(\mathrm{ml}/\,\min /1{{\rm{.73m}}}^{{\rm{2}}})={\rm{194}}\times {\rm{serum}}\,{{\rm{Cr}}}^{-{\rm{1.094}}}\times {{\rm{age}}}^{-{\rm{0.287}}}({\rm{for}}\,{\rm{female}})\times {\rm{0.739}},$$

where Cr = serum creatinine level (mg/dl).

Subjects were categorized into CKD stages on the basis of their eGFR and proteinuria in accordance with JSN and KDIGO CKD guidelines^1,6^. Because subjects in CKD stages G4 and G5 were very rare in this study, they were categorized into G3b. In this study, CKD stages were shown as G stages from G1 to G3b with P from P(−) to P(2+). Hypertension was defined as having a systolic blood pressure of ≥140 mmHg or a diastolic blood pressure of ≥90 mmHg, or being on antihypertensive medication^17^. DM was defined as having a casual blood glucose level of ≥200 mg/dL or a high HbA1c level of ≥6.5%, or being on antidiabetic medication^18^. Dyslipidemia was defined as having a serum LDL level of ≥140 mg/dL or being on lipid-lowering medication^19^.

### Statistical analyses

Normally distributed variables are presented as mean ± standard deviation (SD). The distribution of CKD stages was evaluated by heat mapping (Supplementary Fig. S6). Each subject’s CKD stage can be treated as a position coordinate; for example, G1 P(−) is (0, 0) (Supplementary Fig. S7A). Here, one CKD-stage progression of G is expressed (1, 0), and that of proteinuria is (0, 1). For example, given the CKD stage in 2009 and in 2016 being (G_2009_, P_2009_), and (G_2016_, P_2016_), respectively, the change in CKD stage from 2009 to 2016 can be treated as a vector, (G_2016_ - G_2009_, P_2016_ - P_2009_) (Supplementary Fig. S7B). The mean vector of subjects at each CKD stage in 2009 indicated the trend of changes in CKD stage.

CKD stage is classified on the basis of the KDIGO prognostic categories of CKD, namely, low risk, moderately increased risk, high risk, and very high risk of the risk of ESRD, CVD, and death^6^. Here, the outcome was defined as the progression of a prognostic category of CKD (from 2009 to 2016) or the high risk at the end of this study (2016).

Bayesian network is a kind of probabilistic graphical model that shows variables and their causal relationships via a directed acyclic graph, and represents the probabilistic relationships between variables. The Bayesian network was used to evaluate the relationships between the outcome and the variables using two points of time-series data (2009 and any of the following year from 2010 to 2015). The incremental association Markov blanket method was used for the structure learning algorithm for the Bayesian network. The resulting directed acyclic graph was interpreted as the causal Bayesian network using boot strapping method to average the networks. Continuous variables were discretized using the following cutoff levels determined using receiver operating characteristic curves for the prediction of the outcome using the data in 2009: age, 46 years; BMI, 22.8 kg/m^2^; and waist circumference, 81.4 cm.

SVM is a discriminative classifier defined by a separating hyperplane, which can treat non-linear borderlines, evaluate the effects of variables, and predict the possibilities of the outcome. SVM models including the prognostic categories of CKD were used in this study. Two-thirds of a dataset was used as the training dataset and the remaining one-third was used as the test dataset. In the training dataset, classification was examined on the basis of the three-fold cross validation method, and the accuracy of the prediction was estimated by taking the coverage of three results. Then, using the test dataset, we evaluated the accuracy of the prediction using the SVM models. Using the Gaussian radial basis function kernel, we applied C-support vector classification with variables. These analyses were conducted using SAS version 9.4 (SAS, Inc., NC, USA) and R version 3.5.1 (R project for Statistical Computing, Vienna, Austria). Statistical significance was defined as *p* < 0.05.

## Supplementary information

Supplementary figures

## Data Availability

The datasets generated during and/or analyzed during the current study cannot be publicly available because they are owned by Yamagata Municipalities Mutual Aid Association and Sports Medical Research Center, Keio University. Please ask Sports Center of Keio University about data availability (http://sports.hc.keio.ac.jp/ja/).
