# Supplementary material for: Identifying progressive CKD from healthy population using Bayesian network and artificial intelligence: A worksite-based cohort study
Source: Sci Rep. 2019 Mar 25;9:5082. doi: 10.1038/s41598-019-41663-7 (PMC6434140; doi:10.1038/s41598-019-41663-7)
Supplement: Supplementary file 1 — Supplementary figures [file 41598_2019_41663_MOESM1_ESM.pdf]

Identifying progressive CKD from healthy population using Bayesian network and artificial intelligence: A worksite-based cohort study

Eiichiro Kanda [1], Yoshihiko Kanno [2], Fuminori Katsukawa [3]

1. Medical Science, Kawasaki Medical School, Okayama, Japan.
2. Department of Nephrology, Tokyo Medical University, Tokyo, Japan.
3. Sports Medical Research Center, Keio University, Kanagawa, Japan.

Supplementary Figure S1. Changes in distribution of CKD stages from 2009 to 2010. Page 3.

Supplementary Figure S2. Change in distribution of CKD stages from 2009 to 2012. Page 4.

Supplementary Figure S3. Bayesian network constructed using the data in 2010. Page 5.

Supplementary Figure S4. Explanation of heat map for predicting the outcome from 2009 to 2016. Page 6.

Supplementary Figure S5. Study population. Page 8.

Supplementary Figure S6. Example of heat map. Page 9.

Supplementary Figure S7. Position-coordinate expression of CKD stage. Page 10.

2010 \ 2009		G1				G2				G3a				G3b			
		(−)	(±)	(1+)	(2+)	(−)	(±)	(1+)	(2+)	(−)	(±)	(1+)	(2+)	(−)	(±)	(1+)	(2+)
G1	(−)	2708	120	42	2	775	62	7	4	1	0	0	0	0	0	0	0
	(±)	139	21	9	0	20	4	5	1	0	0	0	0	0	0	0	0
	(1+)	32	2	6	2	4	3	1	0	0	0	0	0	0	0	0	0
	(2+)	3	1	1	6	0	0	0	0	0	0	0	1	0	0	0	0
G2	(−)	872	35	13	1	5969	324	95	12	115	13	3	0	2	0	0	0
	(±)	54	8	0	1	313	81	23	7	3	2	2	1	0	1	0	0
	(1+)	13	1	2	1	55	12	25	13	2	0	0	2	0	0	0	0
	(2+)	3	0	3	2	10	5	6	10	0	0	0	1	0	0	0	0
G3a	(−)	0	0	0	0	166	9	6	0	180	13	4	1	4	1	0	0
	(±)	0	0	0	0	14	1	1	1	8	5	3	1	0	0	0	0
	(1+)	0	0	0	0	5	1	0	0	1	0	2	0	0	0	0	0
	(2+)	0	0	0	0	2	0	4	1	0	1	4	6	0	0	0	0
G3b	(−)	0	0	0	0	1	0	0	0	0	1	0	0	6	0	0	0
	(±)	0	0	0	0	0	0	0	0	0	0	0	0	3	0	1	0
	(1+)	0	0	0	0	0	0	0	0	0	0	0	0	0	0	1	1
	(2+)	0	0	0	0	0	0	0	1	0	0	0	0	0	1	0	7

Supplementary Figure S1. Changes in distribution of CKD stages from 2009 to 2010

The distribution was analyzed using data of subjects with CKD stages in 2009 and 2010 (n=12566). Values show the number of subjects by CKD stage. G1 to G3b and (-) to (2+) are GFR categories of CKD stages, and proteinuria grades, respectively.
Abbreviations: CKD, chronic kidney disease; GFR, glomerular filtration rate.

2012 \ 2009		G1 (-)	G1 (±)	G1 (1+)	G1 (2+)	G2 (-)	G2 (±)	G2 (1+)	G2 (2+)	G3a (-)	G3a (±)	G3a (1+)	G3a (2+)	G3b (-)	G3b (±)	G3b (1+)	G3b (2+)
G1	(-)	1496	81	22	2	403	43	9	0	1	0	0	0	0	0	0	0
	(±)	53	11	8	0	16	6	1	1	0	0	0	0	0	0	0	0
	(1+)	10	2	2	1	3	4	4	0	0	0	0	0	0	0	0	0
	(2+)	5	0	0	5	0	0	1	1	0	0	0	0	0	0	0	0
G2	(-)	1926	65	26	4	5422	342	97	18	78	6	5	0	2	1	0	0
	(±)	94	18	12	0	238	41	14	4	3	0	2	0	0	0	0	0
	(1+)	17	1	3	1	48	8	16	6	0	0	0	1	0	0	0	0
	(2+)	2	1	1	2	3	4	4	4	1	0	0	0	0	0	0	0
G3a	(-)	4	0	0	0	409	15	9	1	177	12	5	2	2	0	0	0
	(±)	1	0	0	0	32	1	3	0	8	3	2	1	0	0	0	0
	(1+)	0	0	0	0	4	1	3	2	0	1	2	2	0	0	0	0
	(2+)	0	0	0	0	1	0	3	0	1	0	0	3	0	0	0	0
G3b	(-)	0	0	0	0	4	0	0	0	2	2	1	0	5	0	0	1
	(±)	0	0	0	0	0	0	0	0	1	0	1	0	2	0	0	0
	(1+)	0	0	0	0	0	0	0	0	0	0	0	0	0	0	0	3
	(2+)	0	0	0	0	0	0	1	1	0	1	0	2	0	1	0	2

Supplementary Figure S2. Change in distribution of CKD stages from 2009 to 2012

The distribution was analyzed using data of subjects with CKD stages in 2009 and 2012 (n=11472). Values show the number of subjects by CKD stage. G1 to G3b and (-) to (2+) are GFR categories of CKD stages, and proteinuria grades, respectively. Abbreviations: CKD, chronic kidney disease; GFR, glomerular filtration rate.

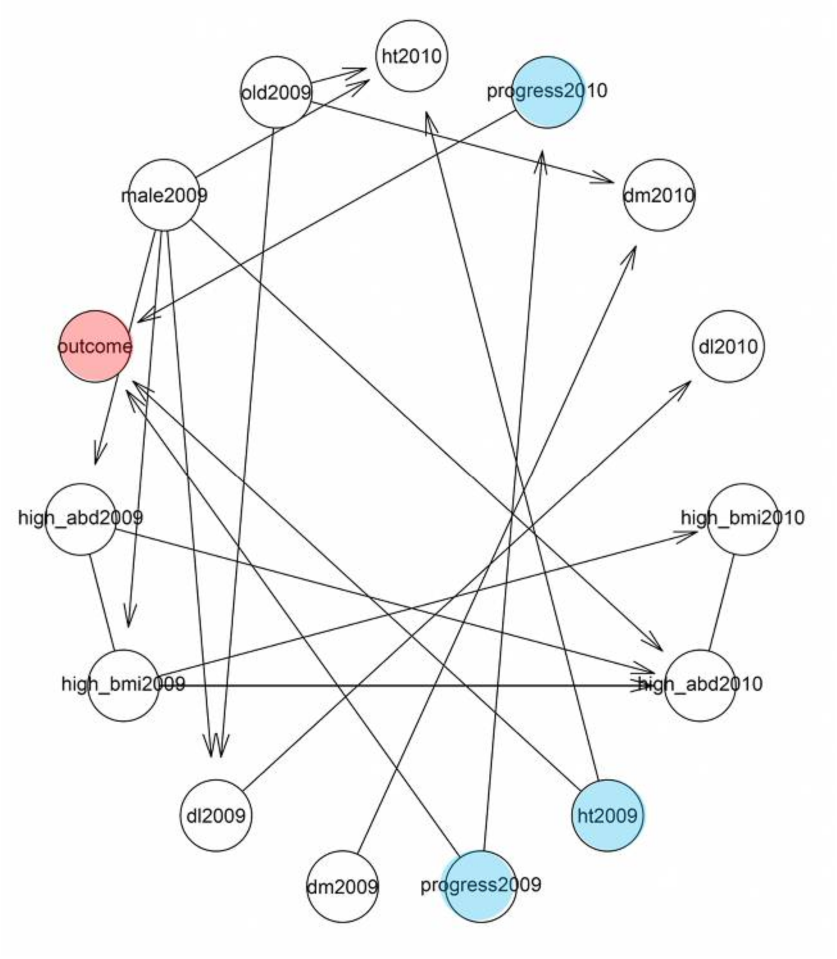

Supplementary Figure S3. Bayesian network constructed using the data in 2010

Arrows show the causal relationships between variables.

Abbreviations: Outcome, the progression of the prognostic category of CKD or very high risk in 2016; progress2009, the prognostic category of CKD in 2009; ht2009, hypertension in 2009; dm2009, diabetes mellitus in 2009; dl2009, dyslipidemia in 2009; old2009, age of 46 years or more in 2009; high_BMI, body mass index of 22.8 kg/m^2 or more in 2009; high_abd, waist circumference of 81.4 cm or more in 2009.

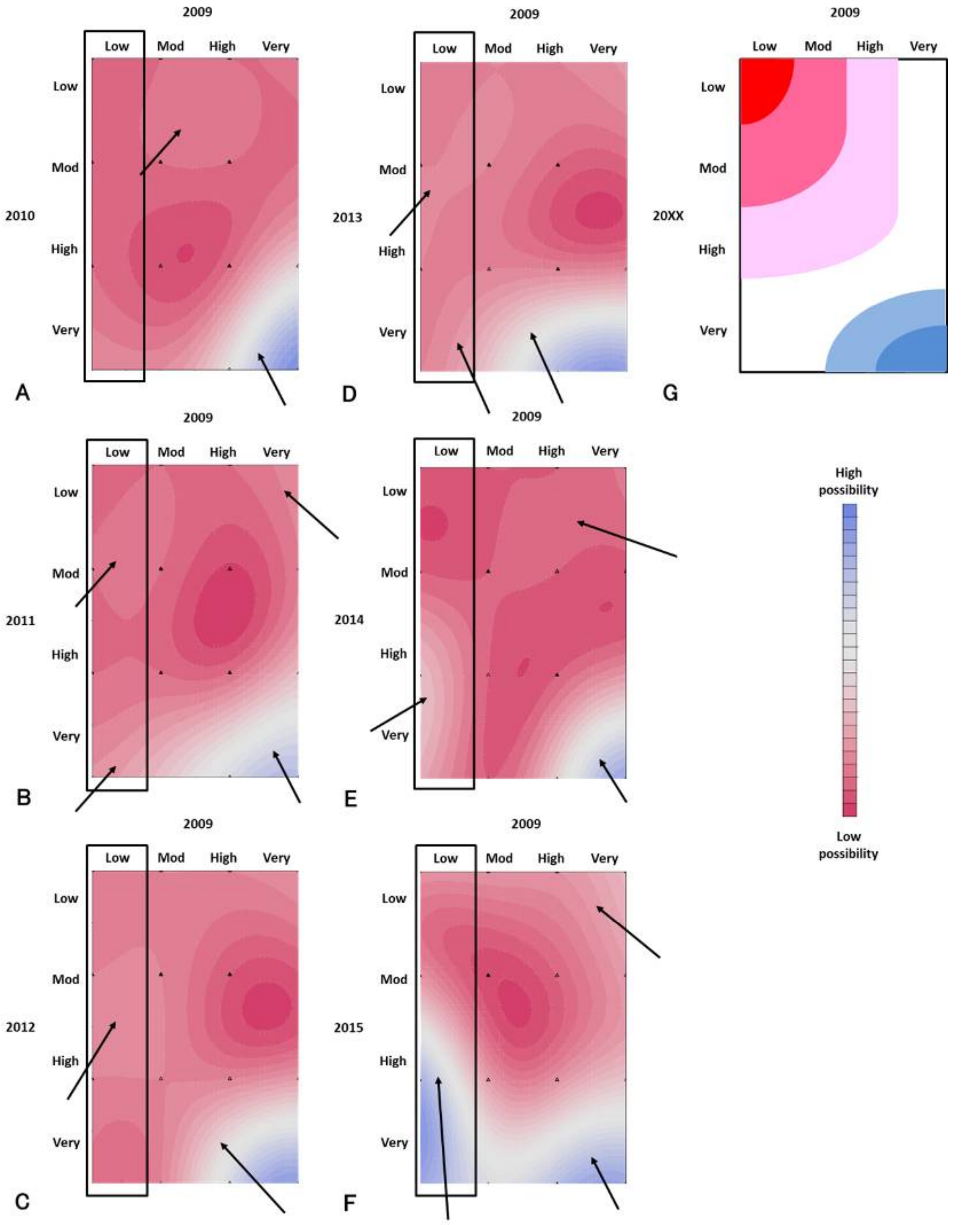

Supplementary Figure S4. Explanation of heat map for predicting the outcome from 2009 to 2016

A heat map shows the possibility of the outcome estimated using SVM models on the basis of data at two points in 2009 and any of the following year. Blue and red areas indicate high and low risks, respectively. Arrows show the high-possibility area of the

outcome. Black boxes show the area for subjects at low risks in 2009. SVM models show that among subjects at low risks in 2009, some of them showed a high possibility of the outcome. Fig. G shows an ideal distribution of the possibility of the outcome. Subjects showing low risks in 2009 and 20xx are expected to have a low possibility of the outcome, and subjects showing very high risks in 2009 and 20xx are also expected to have very high possibilities of the outcomes (Fig. G). However, the distributions of the probabilities in Figs. A to F are different from that in Fig. G.

A. Data 2009 and 2010

B. Data 2009 and 2011

C. Data 2009 and 2012

D. Data 2009 and 2013

E. Data 2009 and 2014

F. Data 2009 and 2015

G. Predicted ideal distribution of possibility in 2009 and 20xx

Abbreviations: Low, low risk of the prognostic categories of CKD; Mod, moderately increased risk; High, high risk; Very, very high risk.

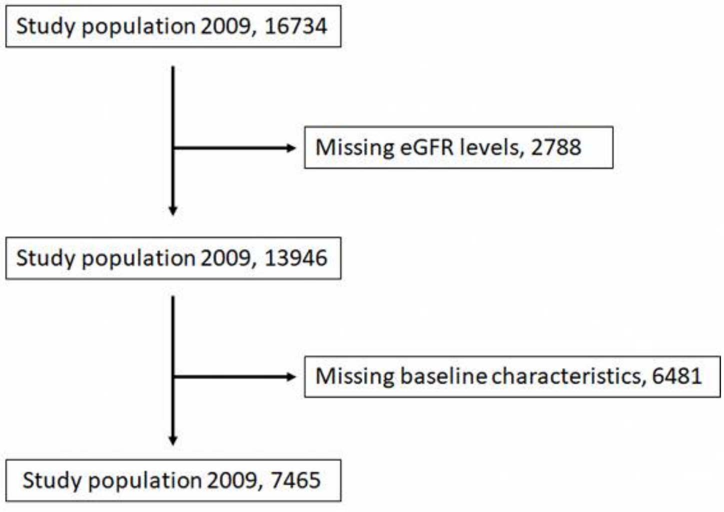

Supplementary Figure S5. Study population

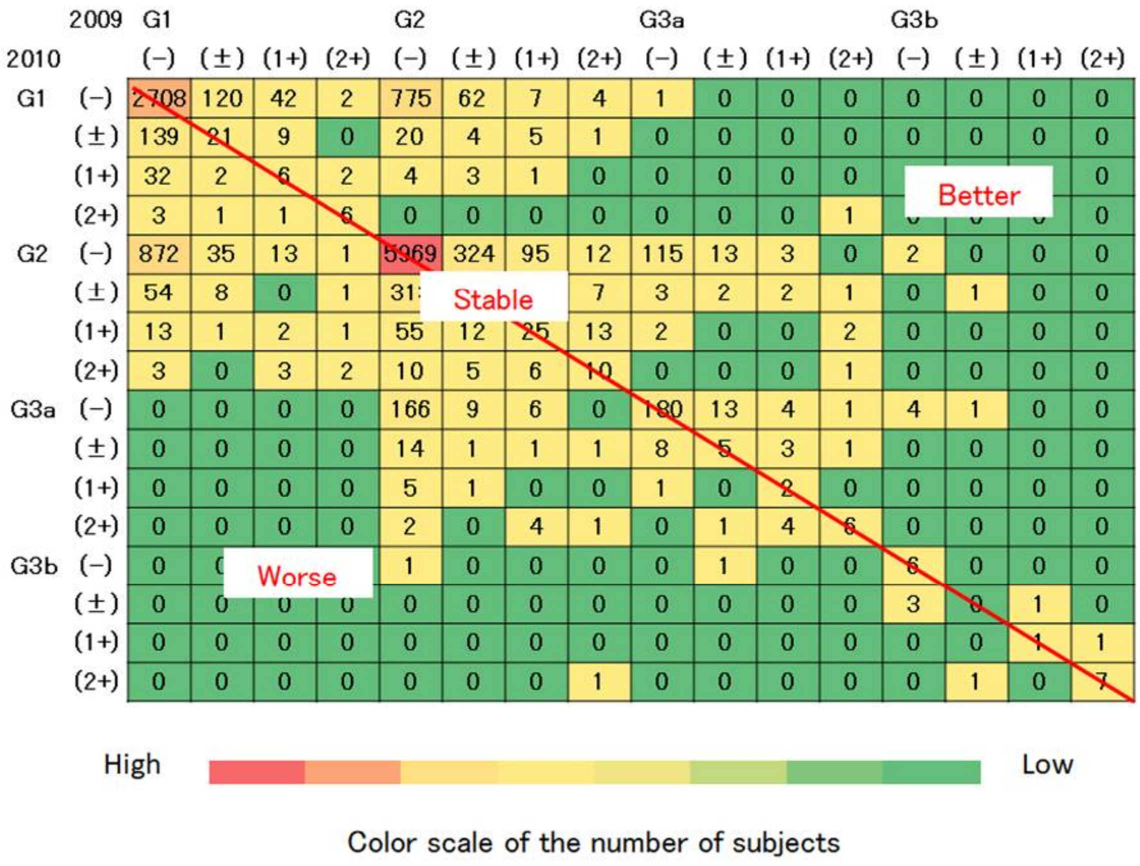

Supplementary Figure S6. Example of heat map

This figure is an example of a heat map of changes in the distribution of CKD stages of participants from 2009 to 2010. Values show the numbers of subjects in various CKD stages. G1 to G3b and (-) to (2+) are GFR category of CKD stage, and proteinuria grades, respectively. The top of the figure shows the CKD stage of a subject in 2009. The left side of the figure shows the CKD stage of a subject in 2010. A cross point shows the number of subjects, whose CKD stage changed from 2009 to 2010.

The kidney function of the subjects in the categories along the red line showed no change. In the categories in the area above and below the red line, the kidney function of the subjects in 2010 was better and worse than those in 2009, respectively.

Abbreviations: CKD, chronic kidney disease; GFR, glomerular filtration rate.

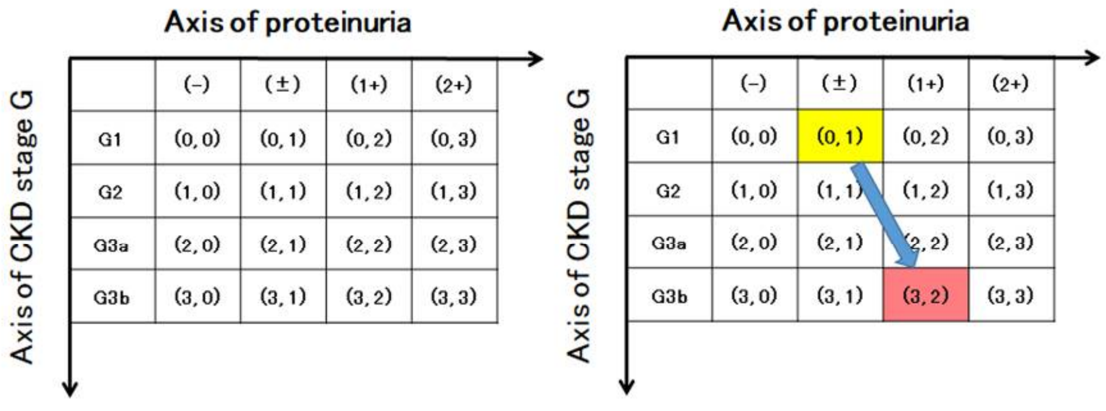

A B

Supplementary Figure S7. Position-coordinate expression of CKD stage

A. Position-coordinate expression of CKD stage

B. Vector expression of change in CKD stage

CKD stage is expressed as a position coordinate. A change in the position coordinate of a subject can be treated as a vector. For example, when a subject's position coordinate changes from (0, 1) to (3, 2), the vector is (3, 1).
